# Effects of Extruder Die Head Temperature and Black Soldier Fly (*Hermetia illucens*) Larvae Meal Inclusion on the Physical Properties of Extruded Asian Seabass Feed Pellets

**DOI:** 10.3390/insects17080759

**Published:** 2026-07-24

**Authors:** Shasha Liu, Murni Marlina Abd Karim, Yong Meng Goh, Qiyou Xu, Zongsheng Qiu, Clement Roy de Cruz

**Affiliations:** 1 International Institute of Aquaculture and Aquatic Sciences, Universiti Putra Malaysia, Port Dickson 71050, Negeri Sembilan, Malaysia; 15550783695@163.com (S.L.);; 2 Department of Aquaculture, Faculty of Agriculture, Universiti Putra Malaysia, Serdang 43400, Selangor, Malaysia; 3 Department of Veterinary Preclinical Science, Faculty of Veterinary Medicine, Universiti Putra Malaysia, Serdang 43400, Selangor, Malaysia; 4 School of Life Sciences, Huzhou Normal University, Huzhou 313000, China; xuqiyou@sina.com

**Keywords:** black soldier fly larvae meal, extrusion, die head temperature, Asian seabass, fishmeal replacement, physical properties

## Abstract

Black soldier fly (*Hermetia illucens*) larvae meal (BSF meal) has emerged as a promising sustainable protein source in aquafeed formulation. This study examined the effects of graded BSF meal inclusion (0%, 20%, 40%, 60%, 80%, and 100% fishmeal replacement) and die head temperature (80 °C, 100 °C, and 120 °C) on the physical properties of extruded diets for Asian seabass (*Lates calcarifer*), with particular attention to their interactive effects. Both factors significantly affected most pellet quality parameters (*p* < 0.05), with the exception that no significant interaction effect was observed for moisture content (*p* = 0.2817). PDI increased progressively with BSF meal inclusion, peaking at 99.14% at 100% substitution and significantly exceeding the control. Higher BSF meal inclusion levels were associated with reduced expansion ratio (ER), bulk density (BD), and sinking velocity (SV). Notably, pellets produced at 80–100% BSF meal inclusion combined with moderate die temperatures (80–100 °C) achieved complete buoyancy (SV = 0.00 m/s). Processing at 80 °C yielded the highest ER and water absorption index (WAI), while 120 °C was associated with elevated water solubility index (WSI), indicating a higher risk of nutrient leaching. These results suggest that BSF meal can replace fishmeal at up to 100% without compromising pellet durability. Extrusion of BSF-based diets at 60–100% inclusion and 80–100 °C is recommended to achieve an optimal balance of structural integrity, hydration properties, and buoyancy for Asian seabass feeds.

## Simple Summary

As global aquaculture continues to expand, the fish industry faces growing pressure to find sustainable and affordable alternatives to fishmeal, a widely used but increasingly scarce and expensive feed ingredient derived from wild-caught fish. Black soldier fly larvae have emerged as a promising solution, as these insects can be reared on organic waste and converted into a nutrient-rich protein ingredient at low environmental cost. However, it remains unclear whether feeds containing high levels of black soldier fly larvae meal can be processed into physically stable pellets suitable for feeding farmed fish. This study investigated how replacing fishmeal with black soldier fly larvae meal at different levels, combined with different processing temperatures, affected the physical quality of extruded feed pellets produced for Asian seabass, a commercially important fish species widely farmed across Southeast Asia. The results showed that increasing black soldier fly larvae meal inclusion did not weaken pellet quality; on the contrary, pellets became more durable and tended to float longer in water, which is beneficial for surface-feeding fish. These findings suggest that black soldier fly larvae meal can be incorporated into fish feeds at high levels without compromising feed quality, supporting its potential as a sustainable fishmeal replacement in commercial aquaculture.

## 1. Introduction

Aquaculture has emerged as the world’s fastest-growing food production sector, currently contributing more than half of global fish consumption [1]. Among commercially cultured species, Asian seabass (*Lates calcarifer*) is of considerable economic importance in Southeast Asia and Australia due to its rapid growth performance, high market value, and adaptability to intensive aquaculture systems [2]. Fishmeal has historically been the primary protein source used to satisfy the nutritional requirements of this carnivorous species due to its excellent digestibility, balanced amino acid profile, and high palatability, which closely match the natural feeding preferences of carnivorous fish [3,4]. However, global fishmeal supplies have remained constrained by the biological limits of marine capture fisheries, while aquaculture-driven demand continues to escalate [5]. The resulting supply–demand imbalance has driven fishmeal prices to persistently elevated levels and introduced considerable volatility into feed production costs, threatening the long-term economic viability of intensive aquaculture operations [3].

Among the candidate alternative protein sources under investigation, black soldier fly (*Hermetia illucens*) larvae meal (BSF meal) has attracted considerable scientific and commercial interest. The BSF larvae are highly efficient bioconverters of organic waste streams, enabling production systems that support circular bioeconomy principles through the conversion of low-value substrates into high-quality protein biomass [6,7]. The nutritional profile of BSF meal is well suited to carnivorous aquaculture species, as it contains 40–60% crude protein on a dry matter basis, a favourable amino acid composition that reasonably resembles that of fishmeal, moderate lipid levels enriched with lauric acid that has been associated with antimicrobial properties, and appreciable amounts of minerals, including calcium and phosphorus [8]. Feeding trials conducted across various fish species have demonstrated that partial replacement of fishmeal with BSF meal can maintain satisfactory growth performance, feed conversion efficiency, and physiological health indicators, supporting its potential as a sustainable alternative protein source in commercial aquafeeds [9].

Importantly, however, the successful incorporation of BSF meal into commercial aquafeed formulations depends not only on its nutritional suitability. Modern aquafeeds for carnivorous species such as Asian seabass are predominantly produced using single-screw or twin-screw extrusion technology, a thermomechanical process in which raw feed mixtures are transformed into structured pellets through the combined effects of heat, pressure, and mechanical shear [10]. The physical quality attributes of extruded pellets, including expansion ratio, bulk density, moisture content, pellet durability, and water stability, are key determinants of feed intake efficiency, nutrient retention, waste output, and overall water quality in aquaculture production systems [11]. Pellets formulated for Asian seabass, a surface to mid-water feeder, must additionally maintain adequate buoyancy and mechanical integrity throughout the feeding period to ensure efficient feed utilization and minimize environmental loading.

The physicochemical composition of BSF meal differs substantially from that of fishmeal in ways that are mechanistically relevant to extrusion behaviour. The BSF meal contains appreciable levels of chitin, a structural polysaccharide derived from insect exoskeletons, as well as a distinct lipid profile and different protein solubility characteristics. These compositional differences are expected to influence starch gelatinization dynamics, protein network formation, and melt viscosity within the extruder barrel, thereby affecting pellet expansion, texture, and structural cohesion in ways that cannot be predicted solely from nutritional composition [11,12]. Several studies have reported that high insect meal inclusion levels compromise pellet durability and water stability, raising practical concerns regarding the technological suitability of BSF-based diets at commercially relevant inclusion rates [13,14]. Moreover, die head temperature, a critical and directly controllable extrusion parameter, regulates the extent of protein denaturation, starch conversion, and pellet expansion; however, the interactive effects of BSF meal inclusion level and die head temperature on pellet quality remain poorly understood under extrusion conditions, and a systematic evaluation linking compositional characteristics to processing behaviour and final pellet properties is still lacking.

The present study was therefore designed to systematically evaluate the combined effects of graded BSF meal substitution levels (0%, 20%, 40%, 60%, 80%, and 100% replacement of fishmeal) and die head temperatures (80 °C, 100 °C, and 120 °C) on the physical quality attributes of extruded pellets, namely moisture content, expansion ratio, bulk density, pellet durability index, water absorption index, water solubility index, and sinking velocity. By characterising the interactive effects of BSF meal inclusion and processing temperature on pellet quality, this study aims to provide an evidence-based framework for optimising extrusion conditions in the production of BSF-based aquafeeds for Asian seabass, thereby supporting the transition towards more sustainable and economically resilient aquaculture feed systems.

## 2. Materials and Methods

### 2.1. Ingredient Preparation

Defatted black soldier fly (*Hermetia illucens*) larvae meal was purchased from Life Origin Sdn. Bhd. (Seri Kembangan, Selangor, Malaysia), where larvae were reared on standardized organic substrates to ensure nutrient consistency. Fishmeal was utilized as the primary protein source for the control group and was partially substituted by BSF meal in the experimental treatments. Tapioca starch was included as the main carbohydrate and binding agent. All dry ingredients were finely ground to pass through a 250 μm mesh sieve to ensure a uniform particle size, which is critical for stable extrusion performance and consistent pellet quality. The proximate compositions of the primary protein sources (BSF meal and fishmeal) are presented in Table 1.

### 2.2. Experimental Diet Design

Six experimental diets were formulated to be isonitrogenous and isolipidic, with crude protein and crude lipid contents maintained at approximately 45% and 12%, respectively (Table 2). The basal diet consisted of soybean meal, shrimp meal, poultry by-product meal (PBM), corn meal, and essential feed additives, including lipid sources, vitamin premix, and mineral premix. Defatted black soldier fly larvae meal was used to replace fishmeal at six inclusion levels (0%, 20%, 40%, 60%, 80%, and 100%).

For diet preparation, all dry ingredients were first finely ground and weighed according to the formulation. The ingredients were then mixed thoroughly using a dough mixer to ensure uniform distribution. After dry mixing, lipid sources were added evenly into the mixture. Water was gradually incorporated until the moisture content reached approximately 40%, which was suitable for the extrusion process. The prepared mixtures were subsequently packed in polyethylene bags and kept overnight in an air-conditioned room to allow the moisture to distribute evenly before extrusion.

### 2.3. Extrusion Process

Following a 15 min thorough mixing of the dry ingredients in a dough mixer, deionized water was gradually incorporated to reach a pre-extrusion target moisture content of approximately 40%. The moistened mixture was manually fed into the hopper and then processed through a single-screw extruder (Brabender KE19, Brabender GmbH, Duisburg, Germany) equipped with a 3 mm die. To facilitate optimal protein denaturation and starch gelatinization, the extruder was operated at a screw speed of 300 rpm. The barrel consisted of two fixed heating zones maintained at 78 °C and 80 °C, while the die head temperature was independently set at 80 °C, 100 °C, or 120 °C according to the experimental treatment. Each of the 18 treatment combinations was independently extruded three times, with each extrusion run representing one process replicate. Representative pellet samples were collected separately from each process replicate for subsequent physical properties analyses. Following extrusion, the pellets were dried in a forced-air oven at 60 °C for 16 h, cooled to room temperature, and stored in airtight containers at −20 °C for subsequent physical and chemical analyses [15].

### 2.4. Physical Properties Analysis

Pellet physical properties were evaluated to determine the technological suitability of BSF meal inclusion in extruded aquafeeds [16].

#### 2.4.1. Moisture Content (MC)

The moisture content of the extruded pellets was determined using the oven-drying method. From each replicate, three samples were dried in a forced-air oven at 120 °C until a constant weight was achieved. The moisture content (%) was calculated as follows:

$$\mathrm{MC}\;\left(\%\right)=\frac{W_{\mathrm{initial}}-W_{\mathrm{final}}}{W_{\mathrm{initial}}}\;\times \;100$$

where W_initial_ and W_final_ represent the weight of the sample before and after drying, respectively.

#### 2.4.2. Expansion Ratio (ER)

The expansion ratio of the extruded pellets was determined by measuring the diametrical expansion relative to the extruder die orifice. Pellet diameter was determined following the method described by Conway and Anderson [17]. From each replicate, ten intact pellets were randomly selected from each replicate for diameter measurement using a digital caliper (ABSOLUTE Series No. 500, Mitutoyo Co., Tokyo, Japan). The ER was calculated according to the following equation:

$$\mathrm{ER}\;=\;\frac{D_{p}}{D_{d}}$$

where D_p_ is the diameter of the extruded pellet (mm) and D_d_ is the die diameter (3 mm).

#### 2.4.3. Bulk Density (BD)

Pellets from each replicate were poured into a tared 100 mL measuring cylinder up to the 100 mL mark. The weight of the pellets was recorded using an analytical balance. To standardize the method, the cylinder was not tapped prior to weighing [18]. The bulk density was calculated as follows:

$$\mathrm{BD}\left(\frac{g}{L}\right)=\frac{\mathrm{Weight}\;\mathrm{of}\;\mathrm{pellets}\;(g)}{100\;\mathrm{mL}}\;\times \;1000$$

#### 2.4.4. Pellet Durability Index (PDI)

The pellet durability index was measured to evaluate the resistance of the extrudates to mechanical stress following modified methods described previously [16,19]. A 50.0 g sample of pellets (P_bt_) from each replicate was placed into a friabilator (DF-3, Distek, North Brunswick, NJ, USA) and rotated at 25 rpm for 10 min. After tumbling, the samples were sieved through a 2 mm mesh for 1 min to remove broken particles and fines. The intact pellets retained on the sieve were then collected and weighed (P_at_). The PDI (%) was calculated according to the following equation:

$$\mathrm{PDI}\;=\;\frac{P_{\mathrm{at}}}{P_{\mathrm{bt}}}\;\times \;100$$

#### 2.4.5. Water Absorption Index (WAI) and Water Solubility Index (WSI)

The water absorption index and water solubility index of the extruded pellets were determined to evaluate their hydration characteristics. Extruded pellets were ground and passed through a 200 μm sieve to obtain a fine powder. A 2.5 g sample of the powder (W_ds_) was transferred into a pre-weighed 50 mL centrifuge tube and dispersed in 30 mL of distilled water. The mixture was incubated in an oven at 30 °C for 30 min, with intermittent vortex mixing performed three times during this period. Subsequently, the mixture was centrifuged at 3000× *g* for 10 min at room temperature. The supernatant was carefully decanted into a pre-weighed aluminum dish to determine the WSI, while the remaining gel sediment in the centrifuge tube was weighed (W_g_) for the WAI calculation. The aluminum dish containing the supernatant was dried in an oven at 135 °C for 2 h to obtain the weight of the dried solids (W_ss_) [16]. The WAI and WSI (%) were calculated using the following equations:

$$\mathrm{WAI}\;=\;\frac{W_{g}}{W_{\mathrm{ds}}}$$

$$\mathrm{WSI}\;\left(\%\right)=\frac{W_{\mathrm{ss}}}{W_{\mathrm{ds}}}\;\times \;100$$

#### 2.4.6. Sinking Velocity (SV)

The sinking velocity (m/s) of the extruded pellets was evaluated using a 1000 mL glass measuring cylinder filled with distilled water at room temperature. The water column height, representing the vertical distance travelled by the falling extrudates, was fixed at 0.34 m. From each replicate, three intact pellets were randomly selected for sinking velocity determination, and the sedimentation time (t, s) was recorded using a digital stopwatch [20]. The sinking velocity was calculated as follows:

$$\mathrm{SV}\;=\;\frac{H}{t}$$

where H is the travel distance (0.34 m) and t is the mean sinking time (s).

### 2.5. Statistical Analysis

The independent extrusion run was considered the experimental unit. Measurements obtained from multiple pellets or analytical subsamples within each process replicate were averaged before statistical analysis. Therefore, the statistical analysis was based on three independent process replicates per treatment combination (*n* = 3), giving a total of 54 experimental units. All data were expressed as mean ± standard deviation (SD). Prior to analysis, data were tested for normality and homogeneity of variances using the Shapiro–Wilk and Levene’s tests, respectively. Two-way analysis of variance (ANOVA) was performed to examine the effects of BSF meal inclusion level, extrusion temperature, and their interaction on the physical properties of the extruded pellets. When significant differences were detected (*p* < 0.05), treatment means were compared using Tukey’s HSD post hoc test. All statistical analyses were conducted using JMP Pro Version 16 (SAS Institute Inc., Cary, NC, USA).

## 3. Results

### 3.1. Pellet Quality

The effects of die head temperature (T), black soldier fly larvae meal (BSF meal) inclusion level, and their interaction (T × BSF) on pellet moisture content (MC), expansion ratio (ER), bulk density (BD), and pellet durability index (PDI) are presented in Table 3. Both T and BSF meal significantly affected all four pellet quality parameters (*p* < 0.0001). The interaction (T × BSF) significantly influenced ER, BD, and PDI (*p* < 0.0001), while no significant interaction effect was observed for MC (*p* = 0.2817).

Among the die head temperatures tested, pellets produced at 100 °C showed the highest MC (3.11%), compared with 80 °C (2.93%) and 120 °C (2.90%), with significant differences observed among temperature levels (*p* < 0.0001). Expansion ratio was highest at 80 °C and lowest at 120 °C, with a significant difference observed between these two temperatures levels. Bulk density was significantly lower at 80 °C (516.16 g/L), while values at 100 °C (527.63 g/L) and 120 °C (527.85 g/L) were similar. Pellet durability index was highest at 80 °C (98.85%) and lowest at 120 °C (98.76%), although all values remained above 98.5% (*p* = 0.0186).

With respect to BSF meal inclusion level, MC increased progressively with increasing BSF meal, ranging from 2.73% at 0% BSF meal to 3.25% at 100% BSF meal (*p* < 0.0001). Expansion ratio decreased with increasing BSF inclusion (*p* = 0.0010), from 1.17 at 0% to 1.14 at 100%. BD generally decreased with increasing BSF meal inclusion, reaching a minimum at 80% BSF meal (513.41 g/L) and a maximum at 0% BSF meal (538.65 g/L) (*p* < 0.0001). In contrast, PDI increased significantly with BSF meal inclusion level, peaking at 100% BSF meal (99.14%) compared to the lowest value at 0% BSF meal (98.50%) (*p* < 0.0001).

### 3.2. Water Absorption Index, Water Solubility Index, and Sinking Velocity

The effects of die head temperature (T), black soldier fly larvae meal (BSF meal) inclusion level, and their interaction (T × BSF) on water absorption index (WAI), water solubility index (WSI), and sinking velocity (SV) are shown in Table 4. All three parameters were significantly affected by T, BSF meal, and T × BSF interaction (*p* < 0.0001), except that WSI showed a less pronounced but still significant response to BSF meal (*p* = 0.0080) and T × BSF interaction (*p* = 0.0054).

With respect to die head temperature, water absorption index decreased with increasing temperature, with the highest value recorded at 80 °C (5.78), followed by 100 °C (4.22) and 120 °C (3.43) (*p* < 0.0001). In contrast, the water solubility index increased across the same temperature range, from 30.41% at 80 °C to 38.57% at 100 °C and 42.93% at 120 °C (*p* < 0.0001). Sinking velocity was significantly higher at 120 °C (0.0276 m/s) than at 80 °C (0.0231 m/s) and 100 °C (0.0232 m/s), which showed similar values (*p* < 0.0001).

Regarding BSF meal inclusion level, water absorption index showed a non-linear response, peaking at 60% BSF meal (5.68) and declining at higher inclusion levels (*p* < 0.0001). Water solubility index did not show a clear trend with increasing BSF meal inclusion, though differences among treatments were significant (*p* = 0.0080), with the highest WSI recorded at 80% BSF meal (41.55%) and the lowest at 40% BSF meal (34.04%). Sinking velocity decreased steadily with increasing BSF meal inclusion, from 0.0469 m/s at 0% BSF meal to 0.0134 m/s at 100% BSF meal (*p* < 0.0001), indicating that higher BSF meal substitution rates were associated with reduced pellet sinking velocity in water. The significant T × BSF interaction indicates that the effects of BSF meal inclusion on WAI, WSI, and SV varied depending on the die head temperature applied during extrusion.

## 4. Discussion

Moisture content (MC) is an important indicator of extruded feed quality, as it influences pellet stability and storage performance. In the present study, MC was significantly affected by both die head temperature and BSF meal inclusion level, whereas no significant interaction effect was observed between the two factors. The higher MC observed at 100 °C may be related to improved water retention within the feed matrix during extrusion, while further increases in temperature may contribute to additional moisture loss during processing. Similar temperature-related changes in MC have also been reported in previous extrusion studies [15]. In addition, MC increased progressively with increasing BSF meal inclusion level, which may be associated with the water-binding properties of chitin and other components present in BSF meal. Comparable increases in pellet moisture content following BSF meal inclusion have been reported by Wrasiati et al. [21]. Overall, the results indicate that both extrusion temperature and BSF meal inclusion level influenced pellet moisture content, although their interaction was not significant under the conditions of the present study.

Expansion ratio (ER) is an important physical property of extruded aquafeeds because it influences pellet structure and buoyancy in water. In the present study, ER decreased slightly with increasing die head temperature and BSF meal inclusion level, although the magnitude of change was relatively small across treatments. A significant interaction effect between extrusion temperature and BSF meal inclusion level was also observed, indicating that the response of pellet expansion depended on both processing condition and feed formulation. The lower ER observed at higher extrusion temperatures may be associated with structural modifications within the feed matrix during extrusion, which can limit pellet expansion after exiting the die. In addition, the gradual reduction in ER with increasing BSF meal inclusion may be related to the partial replacement of starch-rich ingredients by BSF meal components, including chitin and lipids, which could reduce expansion capacity during extrusion. Similar reductions in pellet expansion following insect meal inclusion have been reported in trout and turbot feeds by Borgogno et al. [22].

Bulk density (BD) is an important physical characteristic of extruded aquafeeds, as it is closely related to pellet expansion and buoyancy. In the present study, BD generally decreased with increasing BSF meal inclusion level, which corresponded with the reduction in ER observed across treatments. Similar reductions in BD following insect meal inclusion have also been reported by Sealey et al. [23]. The lower BD values at higher BSF meal inclusion levels may be associated with changes in feed matrix composition caused by the replacement of fishmeal and starch-rich ingredients with BSF meal [24]. In addition, the significant interaction effect between extrusion temperature and BSF meal inclusion level observed in the present study suggests that both processing condition and feed formulation contributed to the final pellet density.

Pellet durability index (PDI) is an important indicator of pellet resistance to mechanical damage during handling and transportation, and values above 95% are generally considered acceptable for commercial aquafeeds [25]. In the present study, all treatments showed high PDI values exceeding 98%, indicating relatively high pellet durability under the conditions of the present evaluation. Although PDI increased slightly with increasing BSF meal inclusion level, all treatments maintained PDI values above 98%, suggesting that BSF meal inclusion did not adversely affect pellet durability in the present formulation. Similar findings were reported by Irungu et al. [13], who also observed consistently high pellet durability (99%) regardless of insect meal inclusion level.

Water absorption index (WAI) reflects the water-holding capacity of extruded pellets and is commonly associated with starch gelatinization during extrusion processing. In the present study, WAI decreased with increasing die head temperature, which may be related to structural changes in starch components at higher extrusion temperatures. Similar temperature-related reductions in WAI have been reported by de Cruz et al. [16]. In contrast, WAI showed a non-linear response to BSF meal inclusion level, increasing at moderate inclusion levels before declining at higher substitution rates. This trend may be associated with the water-binding properties of BSF meal components at moderate inclusion levels, while excessive replacement could reduce the contribution of starch to water absorption during extrusion [26]. In addition, the significant interaction between extrusion temperature and BSF meal inclusion level suggests that pellet hydration properties were influenced by both processing condition and feed formulation.

Water solubility index (WSI) reflects the amount of soluble materials released from the feed matrix during extrusion and is commonly used as an indicator of starch degradation and molecular breakdown [27]. In the present study, WSI increased significantly with increasing die head temperature, indicating greater formation of soluble components at higher extrusion temperatures. Similar temperature-related increases in WSI have been reported in previous extrusion studies involving aquafeeds [16,28]. Although BSF meal inclusion level also significantly affected WSI, no clear increasing or decreasing trend was observed across the substitution levels. This suggests that the influence of BSF meal on pellet solubility may be more complex than that of extrusion temperature alone. In addition, the significant interaction between extrusion temperature and BSF meal inclusion level indicates that the response of WSI was dependent on both feed formulation and processing conditions.

Sinking velocity (SV) is an important physical property of aquafeeds because it influences the time available for fish to detect and consume pellets before they sink below the feeding zone [29]. In the present study, SV decreased markedly with increasing BSF meal inclusion level, indicating improved pellet buoyancy at higher substitution levels. This observation corresponded with the reduction in bulk density observed in the same treatments, suggesting a close relationship between pellet density and sinking behaviour. Similar observations have been reported in studies evaluating insect meal-based aquafeeds, where improved buoyancy was generally associated with lower pellet density [7,29]. In addition, a significant interaction effect between die head temperature and BSF meal inclusion level was detected for SV, indicating that pellet sinking behaviour was influenced by both feed formulation and extrusion conditions. These findings suggest that appropriate combinations of BSF meal inclusion level and extrusion temperature may be used to produce pellets with desirable buoyancy characteristics for Asian seabass culture.

## 5. Conclusions

This study demonstrates that graded substitution of fishmeal with defatted black soldier fly larvae meal significantly influenced the physical quality of extruded diets for Asian seabass, with outcomes shaped by both inclusion level and die head temperature. Contrary to concerns regarding the technological suitability of insect meal in extruded aquafeeds, BSF meal inclusion up to 100% did not compromise pellet durability; rather, PDI improved progressively with increasing substitution. Higher BSF meal inclusion levels were also associated with reduced bulk density and sinking velocity, with complete pellet buoyancy achieved at 80–100% inclusion combined with moderate die temperatures of 80–100 °C. Processing temperature emerged as a critical determinant of hydration properties, with 80 °C yielding superior water absorption and 120 °C promoting elevated water solubility, the latter indicating a potential risk of nutrient leaching under high-temperature conditions. Taken together, these findings indicate that extrusion of BSF-based diets at 60–100% fishmeal replacement and 80–100 °C represents an optimal processing window for producing physically stable, buoyant pellets suitable for Asian seabass aquaculture.

## Figures and Tables

**Table 1:** Proximate composition of fishmeal and black soldier fly meal (% dry matter).

Dietary Component	Fishmeal	Defatted Black Soldier Fly
Dry matter (%)	94.25	95.31
Crude protein (%)	65.04	62.95
Crude lipid (%)	11.5	8.8
Ash (%)	12.3	11.2

**Table 2:** The composition of the test diets (g/100 g as-fed basis).

Ingredients	BSF Meal Levels (%)					
	0	20	40	60	80	100
Fishmeal	35.00	28.00	21.00	14.00	7.00	0.00
Soybean meal	20.00	20.00	20.00	20.00	20.00	20.00
Black soldier fly	0.00	7.23	14.46	21.70	28.93	36.16
Shrimp meal	5.00	5.00	5.00	5.00	5.00	5.00
PBM	8.00	8.00	8.00	8.00	8.00	8.00
Tapioca starch	17.92	17.92	17.92	17.92	17.92	17.92
Methionine	0.50	0.57	0.64	0.71	0.78	0.85
Lysine	0.00	0.00	0.00	0.00	0.00	0.00
Fish oil	7.50	7.67	7.84	8.01	8.17	8.34
Soy Lecithin	1.00	1.00	1.00	1.00	1.00	1.00
Vitamin premix ^a^	1.00	1.00	1.00	1.00	1.00	1.00
Mineral premix ^b^	1.00	1.00	1.00	1.00	1.00	1.00
Vitamin c	0.20	0.20	0.20	0.20	0.20	0.20
Choline chloride	0.50	0.50	0.50	0.50	0.50	0.50
α-cellulose	2.38	1.91	1.44	0.97	0.50	0.03
Analyzed nutrients composition (% dry matter basis)						
Crude protein	39.39	40.06	39.21	40.58	39.44	39.36
Crude lipid	12.48	12.50	12.31	12.50	12.55	12.31
Moisture	2.82	2.87	3.14	3.21	3.13	3.33
Ash	12.85	11.71	10.90	10.77	8.44	6.96

^a^ The vitamin premix consists of ascorbic acid (45 g/kg), myo-inositol (5 g/kg), choline chloride (75 g/kg), niacin (4.5 g/kg), riboflavin (1 g/kg), pyridoxine (1 g/kg), thiamin mononitrate (0.9 g/kg), calcium pantothenate (3 g/kg), retinyl acetate (0.6 g/kg), cholecalciferol (0.08 g/kg), vitamin K (menadione) (1.7 g/kg), α-tocopheryl acetate (500 IU/g) (8 g/kg), biotin (0.02 g/kg), folic acid (0.1 g/kg), vitamin B12 (0.001 g/kg), and cellulose (845.1 g/kg); ^b^ the mineral premix consists of KCl (90 g/kg), KI (0.04 g/kg), CaHPO_4_⋅2H_2_O (500 g/kg), NaCl (40 g/kg), CuSO_4_⋅5H_2_O (3 g/kg), ZnSO_4_⋅7H_2_O (4 g/kg), CoSO_4_ (0.02 g/kg), FeSO_4_⋅7H_2_O (20 g/kg), MnSO_4_⋅H_2_O (3 g/kg), CaCO_3_ (215 g/kg), Mg (OH)_2_ (124 g/kg), Na_2_SeO_3_ (0.03 g/kg), and NaF (1 g/kg).

**Table 3:** Physical quality parameters of extruded diets for Asian seabass as influenced by die head temperature, black soldier fly larvae meal inclusion level, and their interaction.

Experiment Diets					
Die Head Temperature (°C)	BSF Meal (%)	MC (%)	ER	BD	PDI (%)
80	0	2.73	1.19 ^abc^	544.68 ^ab^	98.67 ^defgh^
80	20	2.69	1.22 ^a^	528.32 ^cd^	98.83 ^bcdef^
80	40	3.05	1.17 ^abcde^	497.51 ^g^	98.65 ^efgh^
80	60	2.96	1.18 ^abc^	518.95 ^def^	98.94 ^bcd^
80	80	3.04	1.20 ^ab^	514.73 ^ef^	99.10 ^ab^
80	100	3.14	1.07 ^g^	496.76 ^g^	98.94 ^bcd^
100	0	2.82	1.11 ^fg^	526.70 ^de^	98.11 ^i^
100	20	2.87	1.12 ^abcd^	530.16 ^cd^	98.55 ^fgh^
100	40	3.14	1.18 ^abc^	548.06 ^a^	98.87 ^bcde^
100	60	3.21	1.18 ^abc^	527.86 ^cde^	98.99 ^abc^
100	80	3.20	1.18 ^abc^	512.88 ^f^	99.10 ^ab^
100	100	3.40	1.18 ^abc^	520.11 ^def^	99.24 ^a^
120	0	2.63	1.20 ^ab^	545.55 ^a^	98.74 ^cdefg^
120	20	2.65	1.17 ^fg^	530.46 ^bcd^	98.51 ^gh^
120	40	3.14	1.12 ^efg^	517.19 ^def^	98.43 ^h^
120	60	2.86	1.15 ^cdef^	520.16 ^def^	98.82 ^bcdef^
120	80	2.88	1.13 ^def^	512.61 ^f^	98.83 ^bcdef^
120	100	3.21	1.16 ^bcdef^	541.12 ^abc^	99.25 ^a^
Pooled s.e.		0.064	0.009	2.53	0.054
80		2.93 ^b^	1.17	516.16	98.85
100		3.11 ^a^	1.16	527.63	98.81
120		2.90 ^b^	1.15	527.85	98.76
0		2.73 ^c^	1.17	538.65	98.50
20		2.74 ^c^	1.17	529.65	98.63
40		3.11 ^ab^	1.15	520.92	98.65
60		3.01 ^b^	1.17	522.32	98.92
80		3.04 ^b^	1.17	513.41	99.01
100		3.25 ^a^	1.14	519.33	99.14
ANOVA, Pr > *F*					
T		<0.0001	0.0085	<0.0001	0.0186
BSF meal		<0.0001	0.0010	<0.0001	<0.0001
T × BSF		0.2817	<0.0001	<0.0001	<0.0001

Values are expressed as mean ± SD (*n* = 3). Means within the same column bearing different lowercase letters differ significantly (*p* < 0.05, Tukey’s HSD post hoc test). T = die head temperature; BSF meal = black soldier fly larvae meal; T × BSF = interaction effect between die head temperature and BSF meal; MC = moisture content; ER = expansion ratio; BD = bulk density; PDI = pellet durability index.

**Table 4:** Hydration properties and sinking velocity of extruded diets for Asian seabass as influenced by die head temperature, black soldier fly larvae meal inclusion level, and their interaction.

Experiment Diets				
Die Head Temperature (°C)	BSF Meal (%)	WAI	WSI (%)	SV (m/s)
80	0	4.37 ^def^	33.09 ^bcde^	0.0584 ^a^
80	20	4.45 ^def^	35.58 ^bcde^	0.0400 ^bc^
80	40	6.81 ^ab^	26.61 ^e^	0.00 ^f^
80	60	7.36 ^a^	25.42 ^e^	0.00 ^f^
80	80	5.62 ^bcd^	32.79 ^cde^	0.0234 ^e^
80	100	6.05 ^abc^	28.97 ^de^	0.0168 ^e^
100	0	4.41 ^def^	37.02 ^bcde^	0.0477 ^b^
100	20	4.33 ^def^	35.03 ^bcde^	0.0326 ^cd^
100	40	4.70 ^cde^	36.84 ^bcde^	0.0362 ^c^
100	60	5.39 ^bcde^	38.80 ^bcde^	0.0226 ^e^
100	80	2.12 ^h^	46.67 ^ab^	0.00 ^f^
100	100	4.35 ^def^	37.06 ^bcde^	0.00 ^f^
120	0	2.03 ^h^	41.61 ^abcd^	0.0347 ^c^
120	20	4.07 ^efg^	37.61 ^bcde^	0.0408 ^cd^
120	40	4.50 ^def^	38.68 ^bcde^	0.0247 ^de^
120	60	4.28 ^def^	41.05 ^abcd^	0.0186 ^e^
120	80	2.68 ^gh^	45.19 ^abc^	0.0237 ^e^
120	100	3.03 ^fgh^	53.45 ^a^	0.0234 ^e^
Pooled s.e.		0.28	0.026	0.0016
80		5.78 ^a^	30.41 ^c^	0.0231 ^b^
100		4.22 ^b^	38.57 ^b^	0.0232 ^b^
120		3.43 ^c^	42.93 ^a^	0.0276 ^a^
0		3.60 ^cd^	37.24 ^ab^	0.0469 ^a^
20		4.28 ^bc^	36.07 ^ab^	0.0378 ^b^
40		5.34 ^a^	34.04 ^b^	0.0203 ^c^
60		5.68 ^a^	35.09 ^b^	0.0137 ^d^
80		3.47 ^d^	41.55 ^a^	0.0157 ^d^
100		4.48 ^b^	39.83 ^ab^	0.0134 ^d^
ANOVA, Pr > *F*				
T		<0.0001	<0.0001	<0.0001
BSF meal		<0.0001	0.0080	<0.0001
T × BSF		<0.0001	0.0054	<0.0001

Values are expressed as mean ± SD (*n* = 3). Means within the same column bearing different lowercase letters differ significantly (*p* < 0.05, Tukey’s HSD post hoc test). T = die head temperature; BSF meal = black soldier fly larvae meal; T × BSF = interaction effect between die head temperature and BSF meal; WAI = water absorption index; WSI = water solubility index; SV = sinking velocity.

## Data Availability

The original contributions presented in this study are included in the article. Further inquiries can be directed to the corresponding authors.
